# Applications of Bioactive Strontium Compounds in Dentistry

**DOI:** 10.3390/jfb15080216

**Published:** 2024-07-31

**Authors:** Mohamed Mahmoud Abdalla, Osama Sayed, Christie Ying Kei Lung, Vidhyashree Rajasekar, Cynthia Kar Yung Yiu

**Affiliations:** 1 Paediatric Dentistry, Faculty of Dentistry, The University of Hong Kong, Hong Kong, China; mohamabd@hku.hk (M.M.A.); vidhya@connect.hku.hk (V.R.); 2 Dental Biomaterials, Faculty of Dental Medicine, Al-Azhar University, Cairo 11651, Egypt; 3 Faculty of Dentistry, Fayoum University, Faiyum 63514, Egypt; osamamedhat@outlook.com; 4 Restorative Dental Sciences, Faculty of Dentistry, The University of Hong Kong, Hong Kong, China; cyklung@hku.hk

**Keywords:** strontium, dentistry, radiopacity, regeneration, hypersensitivity, antibacterial

## Abstract

Divalent cations have captured the interest of researchers in biomedical and dental fields due to their beneficial effects on bone formation. These metallic elements are similar to trace elements found in human bone. Strontium is a divalent cation commonly found in various biomaterials. Since strontium has a radius similar to calcium, it has been used to replace calcium in many calcium-containing biomaterials. Strontium has the ability to inhibit bone resorption and increase bone deposition, making it useful in the treatment of osteoporosis. Strontium has also been used as a radiopacifier in dentistry and has been incorporated into a variety of dental materials to improve their radiopacity. Furthermore, strontium has been shown to improve the antimicrobial and mechanical properties of dental materials, promote enamel remineralization, alleviate dentin hypersensitivity, and enhance dentin regeneration. The objective of this review is to provide a comprehensive review of the applications of strontium in dentistry.

## 1. Introduction

Strontium (Sr) belongs to group 2 of the periodic table and has two electrons in its valence shell. It loses two electrons to become Sr^2+^, a divalent cation, after the formation of strontium compounds. These compounds have been used in dental materials for a long time. Strontium was discovered in Strontian, a village in Scotland, in 1790, and successfully isolated in 1808 by Davy [1,2]. With an atomic number of 38 and atomic mass of 87, Sr is classified as an alkaline earth metal in the periodic table, belonging to the same group as calcium (Ca) [3]. It is widely available and constitutes approximately 0.02–0.03% of the Earth’s crust. Due to strontium’s chemical similarity to Ca, fruits and vegetables absorb it from the soil [4]. As a result, the Sr content in our diet reflects the level found in the soil, albeit at a relatively low concentration compared to Ca. For every 1 mg of Sr, there is 125 mg of Ca [5]. The ratio of Ca to Sr in bodily tissues and fluids mirrors that of the dietary intake [6]. Although Sr is not considered an essential element from a biological standpoint [4], it comprises about 320 mg of the human body and is found in trace amounts in teeth [7]. Our daily intake of Sr ranges from 2.1 to 2.4 mg, primarily from food and water sources [8].

In 1870, Papillon made a significant discovery regarding the biological role of Sr through an in vitro study. After feeding Sr to a pigeon, the analysis demonstrated that it can naturally incorporate into bone [9] through ion exchange with Ca [10,11]. The bone-seeking property of Sr led to further investigations into its systemic effects, revealing that, similarly to Ca, Sr has the ability to influence cardiac contractility, modulate parathyroid secretions, trigger uterine contractions, and incorporate into tooth structure [12,13]. Despite the isomorphism of Sr hydroxyapatite and Ca hydroxyapatite, hydroxyapatite crystals show a preference for Ca ions over Sr ions due to the Sr ion being bigger (1.13 Å) than Ca (0.99 Å) [14]. Due to its similarity with Ca, Sr has been used as a substitute in many Ca-containing compounds [14,15,16].

Interestingly, the benefits of Sr have expanded its applications in dentistry. Strontium is commonly used as a radiopacifier in restorative materials [17], an antimicrobial agent in combination with fluoride [18], and for alleviating dentin hypersensitivity when incorporated in some dentifrices [19]. Furthermore, Sr has been found to stimulate the osteo/odontogenic differentiation of mesenchymal stems cell and human dental pulp stem cells [20,21]. In dental materials, Sr is incorporated in various formulations such as Sr oxide, Sr carbonate, Sr chloride, Sr acetate, and Sr fluoride. It is also used as a substitute for Ca in numerous biomaterials.

**Figure 1:**
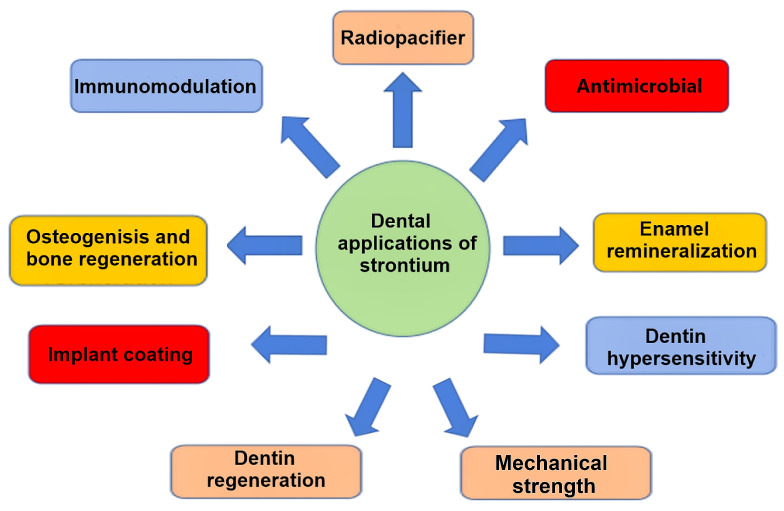
Applications of strontium in dentistry.

Considering the significant benefits of Sr in different dental and biomedical applications and the lack of literature reviews that discuss all the applications of strontium in dentistry (Figure 1), this review aimed to provide a comprehensive overview of the uses and benefits of strontium, the mechanisms of its biological interaction, and its applications in dentistry.

## 2. Radiopacifying Properties of Strontium Compounds

**Figure 2:**
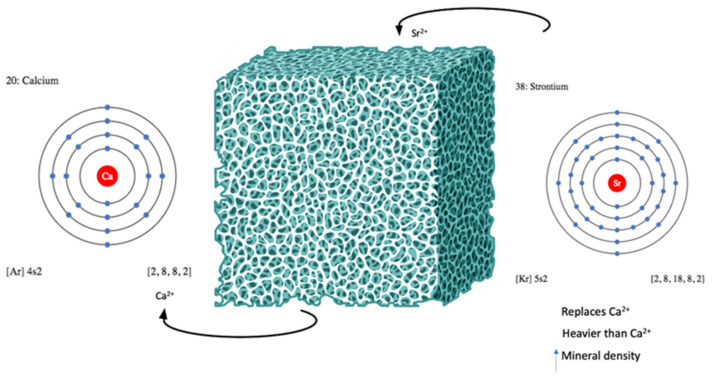
Strontium properties and calcium replacement.

Strontium compounds have been widely used to improve the radiopacity of various materials. Ideally, dental materials should have sufficient radiopacity to be detected by X-ray during dental inspection and distinguished from adjacent carious tooth structure [22]. According to ISO standards and ADA recommendations, the minimal radiopacity of a 1 mm material should be equivalent to 1 mm of aluminum [22,23]. Strontium, a heavy metal with an atomic mass of 87.62 [3], has a higher density (Figure 2) and consequently higher radiopacity when detected by X-ray [24].

Strontium also has a low systemic toxic effect [25], making it an appropriate element for enhancing the radiopacity of many dental materials. Due to its similarity with Ca, Sr has the capability to replace Ca in dental materials [25]. In the 1980s, a patent was filed defining Sr as a radiopacifying agent in glass [26], which is utilized as a filler in dental resin composite restorative materials. In addition, Sr was added to glass ionomer cement to replace some of the Ca and act as a radiopacifying element. It is noteworthy that the incorporation of Sr did not adversely affect the visual opacity of glass ionomer cement [22].

Similarly, incorporating Sr fluoride as a radiopacifying agent in MTA endodontic cement and calcium silicate cement has been shown to significantly enhance radiopacity [24,27]. In a study investigating the impact of incorporating Sr into Biodentine, it was observed that higher radiopacity and compressive strength were achieved with a high fluoride + Sr bioglass modification, although this modification also resulted in an extended setting time [28]. In the context of dental adhesive systems, the inadequate opacity can make it challenging to identify secondary caries radiographically. One study addressed this issue by adding Sr to adhesive resin, which increased the resin’s opacity without significantly affecting the degree of conversion or Young’s modulus [29]. Another study found that the radiopacity of glass ceramics was enhanced with the addition of Sr, attributed to the precipitation of Sr fluorapatite crystals in the structure, instead of calcium fluorapatite, due to the larger ion and higher atomic mass of Sr2+ compared to Ca2+ in the apatite crystal [30]. Additionally, incorporating Sr carbonate into calcium phosphate cement has been shown to significantly improve radiopacity compared to using calcium phosphate alone [31].

## 3. Antimicrobial Effects of Strontium Compounds

Strontium compounds have demonstrated varying antibacterial effects against different bacterial strains, making them suitable for enhancing the antimicrobial properties of medical devices. These compounds can inhibit bacterial growth, impede the permeability of the cytoplasmic membrane, and slow the replication of bacterial chromosomes and cell metabolism [32,33]. In recent years, Sr has been incorporated into dental and orthopedic biomaterials to minimize the risk of secondary caries in dental restorations [34]. It has also been used in injectable bone cement for minimally invasive delivery of antimicrobials in vertebral compression fractures, aiming to inhibit microbial contamination and prevent implant-related infections [35]. Furthermore, Sr-coated implants have been explored for the prevention of peri-implantitis [36].

In dental restorations, the antimicrobial function provided by the restorative material is beneficial in reducing pulpal damage and improving durability of the restoration. Sr-containing glass ionomers have been developed commercially, where Sr ions effectively replace Ca in the composition. In this type of restoration, Sr can be used as a substitute for Ca by incorporating SrO and SrF_2_ instead of CaO and CaF_2_ in the glass-forming mixture [22]. These components are usually insoluble in neutral environments, but in acidic conditions due to oral bacteria, Sr ions are released in greater quantities, exerting antimicrobial effects against bacterial pathogens [37].

Microbiological analysis of secondary caries biofilm has identified that *Streptococcus mutans* and *Actinomyces* are dominant microflora, followed by *porphyromonas gingivalis* [38]. Glass ionomer cements containing Sr exhibited significant antibacterial activity against *Streptococcus mutans* (ATCC 25175) and *Actinomyces viscosus* (ATCC 19246), with Sr contributing particularly to the antibacterial activity against A. viscosus [33]. However, several investigated cements had no detectable antibacterial action against this species. The antibacterial activity of Sr-substituted bioglass on *Porphyromonas gingivalis* and *A. actinomycetemcomitans* was also investigated, and it was found that the antimicrobial activity increased considerably as Sr substitution increased from 0% to 100% [39].

Furthermore, research on injectable Sr-releasing bone cements based on bioglass and polyacrylic acid demonstrated a significant bactericidal effect on *Staphylococcus aureus* (NCIMB6571) and *Streptococcus faecalis* (NCIMB775) [35]. However, Dabsie et al. found that Sr has no inherent antibacterial properties, suggesting that its synergistic reaction with fluoride may enhance the antibacterial activity of dental restorations [40]. The proposed mechanism of antibacterial action of Sr could be due to interference with membrane stability, cellular proteins, and enzymes, as well as the formation of reactive oxygen species that cause severe cell damage [36]. It is also predicted that Sr facilitates fluoride entry by disrupting microbial cell membranes, thereby boosting its effect. Furthermore, as a divalent cation, Sr can alter the composition and structure of dental biofilm by interacting with exopolysaccharides (EPS) [41]. In conclusion, there is no clear consensus on whether Sr has an inhibitory effect on bacteria. However, it is suggested that the Sr2+ ion has a synergistic effect with F- in inhibiting bacterial activity.

## 4. Enamel Remineralization with Strontium Compounds

The remineralization of enamel is a natural, spontaneous healing process primarily based on the deposition of Ca and phosphate ions present in saliva, forming a new layer on the demineralized enamel surface. Modern caries management strategies are designed to prevent and halt the progression of caries [42]. The role of Sr in managing dental caries has been investigated for decades. However, the published data have shown conflicting results regarding whether Sr alone has a remineralizing effect or if its action is synergistic when combined with fluoride [42,43,44,45,46]. The discrepancies in the findings may be attributed to variations in the concentrations and compounds used. Epidemiological research has linked Sr to a lower prevalence of caries [47].

A study conducted by Thuy et al. [48] showed that using a remineralization solution with 10 ppm Sr resulted in the highest rate of mineral gain compared to other Sr concentrations. However, this study did not compare the mineralizing solutions with a control solution without Sr. Similarly, Wang et al. [49] incorporated different Sr concentrations into an acidic solution containing enamel samples and assessed the effect on enamel demineralization. They found that Sr ions at a concentration of 10-2 M could reduce the erosive acidic effect on enamel, leading to less reduction in enamel hardness and phosphorus ion dissolvement compared to acidic solutions without the Sr ions. However, Yassen et al. [47] explored the remineralizing potential of low concentrations of Sr (10, 15 ppm), and found that Sr at a concentration of 10 ppm could remineralize enamel only when combined with fluoride. The same study also suggested that F might be the major cause of remineralization. The integration of fluoride and Sr was proposed to reduce apatite solubility and improve crystallinity in enamel [50].

Another possibility is that carrying fluoride in an uncharged complex with Sr increases the diffusion rate of fluoride through enamel [51]. Studies investigating the remineralizing potential of different concentrations of F alone and in combination with 10 ppm Sr revealed that the enamel remineralization was significantly enhanced with the incorporation of Sr at 0.1 and 0.05ppm F concentrations, suggesting a synergistic enhancement in remineralization due to the interaction between Sr and fluoride [52]. Furthermore, toothpaste containing Sr has been found to increase the Sr content in enamel and reduce enamel solubility, suggesting that the positively charged Sr forms a layer on enamel known as the stern layer, which can mitigate the effects of citric acid [53].

One study substituted Ca in nano-hydroxyapatite with Sr to address issues related to large crystals, acid reactivity, and material strength. This substitution resulted in increased crystallinity and reduced particle size, which facilitated diffusion through small carious lesions and white-spot areas, ultimately leading to improved enamel remineralization [54]. Similarly, Rajendran et al. [55] synthesized Sr-substituted nano-hydroxyapatite in a paste form for better handling and observed enhanced enamel remineralization. Another interesting study by Dai et al. [56] examined the effect of Sr-doped bioactive glass and fluoride on apatite crystal formation during mineralization. The study demonstrated that Sr could replace Ca in the hydroxyapatite crystal lattice, forming an Sr–hydroxyapatite that is bioactive and can bond easily to bony tissues. The formation of two phases, Sr–hydroxyapatite and Sr–fluorhydroxyapatite, was observed, which exhibited greater chemical stability and resistance to dissolution. The study also noted that the larger radius of Sr compared to Ca led to the formation of distorted crystals, but at the low Sr concentration used in their study, this distortion was not observed. The presence of F further enhanced the stability of the structure. The study by Dai et al. highlighted the potential formation and stability of Sr–hydroxyapatite. However, it should be noted that in vitro models do not fully mimic the oral environment. Future research is recommended to determine the exact effect of Sr on remineralization and whether F plays a key role in the process or if Sr can enhance remineralization on its own.

## 5. Managing Dentin Hypersensitivity with Strontium Compounds

Exposed dentinal tubules can cause patients to experience a sharp, short pain in response to external factors such as thermal, chemical, or tactile stimuli. This phenomenon is known as dentin hypersensitivity (DH) and affects nearly one-third of adults globally [57,58]. The use of Sr compounds for managing DH dates back to 1956, when Pawlowska et al. [59] reported the efficacy of a 25% Sr chloride solution in treating DH. They interpreted this result as an indication that Sr salt has a special influence on both the nervous system and the hard tissues, emphasizing its ability to “transform the soft surface” of carious teeth into a hard and smooth structure. Since then, several studies have examined the effectiveness and mechanism of Sr in the treatment of DH.

**Figure 3:**
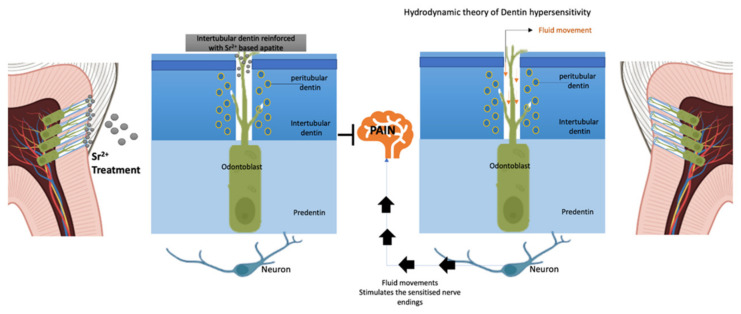
Hydrodynamic theory and mechanism of strontium’s effect on occluding dentinal tubules.

The hydrodynamic theory (shown in Figure 3) proposes a widely accepted explanation for the mechanism of the DH pain mechanism. When adequate stimuli are applied to exposed dentine, the inward or outward flow of dentinal fluids in the tubule network increases, stimulating the baroreceptor nerve fibers in and around the dentine–pulp interface [60]. Histological findings that correlate DH with the percentage of exposed dentinal tubules in sensitive teeth compared to sound teeth support this explanation [61]. To manage DH symptoms, two methodologies are often used. The first is to properly occlude the dentinal tubules to prevent intratubular fluid movement, which has the benefit of a quick onset [62]. The second approach acts on desensitizing the nerve ending by modifying the extracellular potassium concentration to reduce its excitability [63]. Two different approaches have been used to evaluate the effectiveness of Sr in managing DH. The first approach involves assessing the permeability and morphological changes of dentin in vitro. The second approach involves evaluating the pain-relieving effect of Sr in vivo.

Many studies have investigated how Sr works to occlude dentinal tubules and manage DH (Table 1). As an alkaline earth metal element, Sr is expected to have a strong natural absorptive ability for calcified tissues, particularly dentine, due to its high organic composition [64]. It induces precipitations into organic connective tissues, including the odontoblastic processes, to form a sealing film that can occlude open dentinal tubules. This inhibits dentinal fluid movement and helps alleviate DH [65]. Saeki et al. [66] confirmed that Sr can occlude dentinal tubules, as a thick layer containing Sr reaching 20 µm into the tubules was found. Abrasives were not used in the investigation, so the occlusion observed was solely attributable to the impact of Sr. However, this dentinal occlusion was weak and water-soluble, as the layer was undetectable after samples were immersed in DI water. This may explain why Oberg et al. [67] reported that a 10% Sr chloride gel did not show significant dentinal tubular occlusion compared to a negative control group. The analysis was performed after intense rinsing with running distilled water for five minutes.

On the other hand, combining Sr with abrasives in dentifrices has produced a more stable dentinal plug that resists acid and water abrasion, particularly in silica-based products. Olley et al. [68] reported that 8% Sr acetate-based dentifrice in silica base induced dentine tubular occlusion that was resistant to erosive dietary intake with narrower dentine tubules. In contrast, the control paste (1450 ppm sodium fluoride in a silica base) resulted in significantly more open dentinal tubules. Although many studies have found that the occluding materials in theses dentifrices is primarily silica with a few Sr surface deposits [69,70,71,72], the presence of Sr along with silica appears to be related to forming an acid-resistant dentinal occlusion. However, many dentifrices may contain silica-based abrasives and cannot induce dentinal occlusion. This may be due to the absence of sodium lauryl sulfate (SLS) in Sr-based dentifrices, as SLS may compete with silica for attachment to dentine [73].

It is interesting to note that Sr acetate has shown greater clinical effectiveness compared to Sr chloride, and it is also compatible with fluoride and potassium nitrate [74]. However, Dotta et al. [75] conducted a study using strontium carbonate and strontium-substituted calcium carbonate nanoparticles instead of strontium acetate and compared them to calcium carbonate. The hypothesis was that the use of Sr–carbonate nanoparticles would benefit from both the effects of strontium on dentin mineralization and the abrasive properties of carbonates. The study concluded that the performance of the strontium-containing nanoparticles, in terms of tubule obliteration and resistance to acid attack, was better than calcium carbonate and even better than the commercially available product Sensodyne^®^ Rapid Relief. The authors suggested that the selective binding of strontium carbonate and strontium substituted calcium carbonate nanoparticles to the dentin surface is a better explanation for these improved characteristics, rather than simply blocking the exposed dentin tubules and desensitizing the pulp nerve.

Recently, Sr has been incorporated in bioactive glass formulations for the treatment of DH. Bioactive glass is known for its ability to induce the formation of an apatite layer in the presence of body fluid such as saliva [76,77]. It is primarily composed of specific proportions of SiO_2_, Na_2_O, CaO, and P_2_O_5_ [78]. Xia et al. [79] used a toothpaste containing 10 wt. % Sr-substituted Ca phosphate spheres (SCPSs) and found that after one day, the SCPSs had penetrated the dentinal tubules and apatite crystallites had formed on dentin surfaces. After 7 days, the dentin surfaces were completely covered by newly mineralized apatite, and the exposed tubules were fully covered. This freshly mineralized layer consisted of two layers: a porous layer with larger crystals on top and a thick layer beneath. While the porous layer was easily susceptible to mechanical or chemical attacks, the thick layer proved to be more stable [79]. In another study, Acevedo et al. [80] used fluoride varnish as a carrier to apply Sr-incorporated bioactive glass powder on open dentinal tubules. This approach aimed to achieve a synergistic effect in treating DH, resulting in a significant 90% reduction of dentinal permeability.

The efficacy of Sr-containing agents for pain relief in DH is a topic of debate. Martins et al. conducted a review of current clinical research and concluded that Sr is effective only for relieving tactile sensitivity associated with DH [81]. On the other hand, several other reviews (Table 2) have expressed doubts about the significance of Sr in treating DH [82,83,84,85,86,87]. However, a recent randomized clinical trial suggested the combination of photobiomodulation (PBM) with Sr can be effective in treating post-bleaching hypersensitivity. Barros et al. [88] reported that over a one-week period, the combination of PBM and 8% Sr acetate effectively reduced dentin sensitivity compared to imitation PBM + toothpaste without an active ingredient, imitation PBM imitation + toothpaste with 8% Sr acetate, and PBM + toothpaste without the active ingredient [89]. This suggests that the combination of PBM and Sr may have potential in providing relief from dentin hypersensitivity, at least in specific scenarios such as post-bleaching sensitivity.

**Table 1:** In vitro evaluation of Sr efficacy in dentinal tubule occlusion.

Study	Intervention	Outcomes of SEM Analysis
Kodaka et al., 2001 [69]	Sr chloride-based desensitizing toothpaste	91.45% dentinal occlusion after 2 weeks. The occluding material contained artificial silica abrasive within the dentin sludge.
Arrais et al., 2003 [72]	Sr chloride-based desensitizing toothpaste	80.1% dentinal occlusion after 7 days. Deposition of crystal-like structures within the dentinal tubules consisting of Ca carbonate, the abrasive system of the dentifrice.
Banfield et al., 2004 [70]	Sr acetate-based desensitizing toothpaste Sr chloride-based desensitizing toothpaste	90% dentinal occlusion immediately. The occluding material was artificial silica abrasive. >70% dentinal occlusion immediately. The occluding material was artificial silica abrasive.
Oberg et al., 2009 [67]	10% Sr chloride gel	Open and partially obliterated dentin tubules like the no treatment group. Only traces of Sr were detected in the peritubular dentin deposits.
Saeki et al., 2016 [66]	Sr acetate-based desensitizing toothpaste	Clear thin layer of silicon covered the dentine surface and openings of dentine tubules.
	10% Sr acetate solution	Thick Sr-containing layer reaching 20 µm into dentinal tubules. After specimens were soaked in DI water, Sr-containing layer could not be detected.
	Sr chloride-based desensitizing toothpaste	50.54% dentinal occlusion after 7 days. The occlusion material was not reported.

**Table 2:** Systematic reviews evaluating the in vivo efficacy of Sr-containing agents in DH treatment.

Study	Efficacy of Sr-Containing Agents in DH Treatment
Martins et al., 2020 [81]	Sr was effective only for tactile stimulus relief.
Hu et al., 2019 [86]	Similar effects of Sr compared to fluoride, placebo, and potassium-containing toothpastes.
Cruz et al., 2019 [87]	Sr when not combined with potassium was no better than the negative control.
Hu et al., 2018 [85]	Sr when not combined with potassium had no desensitizing activity.
Bae et al., 2015 [84]	There was no statistically significant difference between Sr-containing toothpaste and placebo.
West et al., 2015 [83]	Sr acetate had equivocal pain-relieving effects when compared to arginine and was more effective than fluoride control. There is a lack of high-quality data supporting the use of Sr chloride salts for pain relief in dentine hypersensitivity; additional research is needed to determine whether this salt is useful.
Karim et al., 2013 [82]	There is inadequate evidence to state whether Sr salts per se are effective in reducing DH.

## 6. Dentin Regeneration with Strontium Compounds

The differentiation of human dental pulp stem cells (HDPSCs) into mature odontoblast-like cells is essential for the process of dentin regeneration. Studies have shown that Sr significantly promotes odontogenic differentiation of HDPSCs. However, there have been few investigations on the odontogenic potential of Sr on HDPSCs. Huang et al. [21] reported that Sr chloride elicited the odontogenic differentiation of HDPSCs by modulating the expression and secretion of dentin sialophosphoprotein (DSPP) and dentin matrix protein 1 (DMP-1) in vitro via the CaSR pathway, which shares similarities with osteoblast differentiation. Sr also enhances matrix production and mineralization. The mineralization activity of HDPSCs was further explored using Sr-modified bioglass, where it was found that the alkaline phosphatase activity increased in a dose-dependent manner compared to the control bioglass, while cellular proliferation was inhibited by the Sr-modified bioglass. However, the mechanism behind this inhibition remains unclear.

Abdalla et al. [20] reported that a calcium strontium silicate compound enhanced HDPSC differentiation, setting time, and cytocompatibility compared to calcium silicate alone. Another study conducted by Lee et al. [90] demonstrated an effective increase in the expression levels of genes related to odontogenic differentiation of HDPSCs (collagen type 1 alpha (COL1 A), DSPP, and DMP-1) when treated with a mesoporous bioglass doped with Sr ions (Sr–MBG) compared to non-Sr-containing MBG. Furthermore, Mandakhbayar et al. [91] reported that a Sr-containing nano-bioactive cement (Sr–NBC) enhanced odontogenic differentiation of HDPSCs in vitro and promoted better dentin regeneration in vivo compared to Sr-free NBC. Another study by Basheer et al. [92], which evaluated the effect of Sr-incorporated tetracalcium phosphate cement (STTCP) on HDPSC mineralization potential, discovered that STTCP resulted in a similar improvement in the mineralization and differentiation of HDPSCs to MTA. Considering the significant stimulatory effect of Sr on the odontogenic differentiation of HDPSCs, Sr should be considered in dental biomaterials for pulp regeneration. The enhanced odontogenic differentiation of HDPSCs by Sr is believed to occur through the regulation of Wnt/β-catenin signaling and TGF-β pathways, as well as the regulation of BMP pathway [21].

## 7. Osteogenic Effect and Bone Repair Potential of Strontium Compounds

Bone is one of the key organs in the human body that plays vital roles, such as providing a framework for the muscles, assisting with body movements, protecting the internal organs from physical injuries, and most importantly in the production of blood cells. However, several factors can affect the homeostasis of bone, such as orthopedic trauma caused by accidents, osteoporosis, and other infection-related bone loss, such as osteomyelitis and periodontal disease [93]. Periodontitis is a chronic inflammatory disease that progressively affects the alveolar bone, periodontal ligament, and root cementum. In addition to infection, osteoporosis has also been shown to intensify periodontal bone loss. Although the body has an inherent mechanism to overcome bone defects through endogenous regeneration, in some cases, the natural healing functions are not sufficient to retain the original vitality of the bone [94,95]. Bone tissue engineering is a rapidly growing field that aims to develop materials that can assist or enhance the osteoconductive, osteoinductive, and osteogenic capacity at the site of the bone defect. However, the mechanism of Sr upon osteogenic differentiation is not yet completely understood, and many studies have tried to explore the possible pathways and mechanisms involved in Sr’s effects [96].

**Figure 4:**
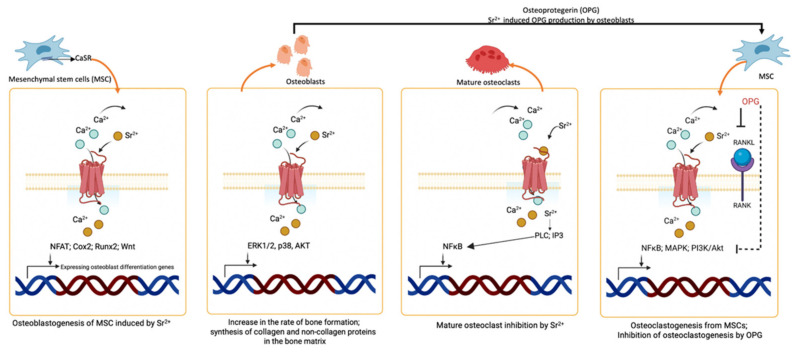
The role of Sr in osteogenesis.

The major components of the bone extracellular matrix (ECM) are collagen types Ⅰ, Ⅲ, and Ⅴ, as well as hydroxyapatite (HA). HA, also known as calcium apatite, contains Ca minerals within the matrix. Sr is an important trace element found in the body and shares similar chemical properties and structure to Ca. As a result, Sr can actively incorporate into the crystal lattice of HA [97,98]. Initially, Sr was suggested as an adjunct for osteoporotic treatment by stimulating bone formation through osteoblasts and suppressing bone resorption by inhibiting the osteoclasts. Although the mechanism by which Sr induced the osteoblasts is not fully understood, the similarities between Ca and Sr suggest that Sr also follows the Ca-sensing receptor (CaSR) pathway. When Sr binds to these receptors, it activates a cascade of pathways, by phosphorylating the PI3K–Akt, Ras–MAPK, and Wnt pathways to enhance the osteogenesis of the osteoblasts and mesenchymal stem cells (MSCs) [99]. Sr also activates the Cn–NFATc pathway, where Sr induces calcineurin to dephosphorylate NFATc (activated state). In the inactive state, this transcription factor remains in the cytoplasm, but upon activation, it is translocated inside the nucleus and promotes the expression of ALP and Runx2 genes to induce osteogenesis [100]. The proliferating osteoblasts then produces M-CSF, and RANKL ligands, which bind with RANK to activate the cascade of signaling pathways, including MAPK, NF-*κ*B, and PI3K–Akt, to stimulate osteoclastogenesis, where osteoclast cells promote bone resorption (Figure 4). The osteoprotegerin (OPG) receptor, belonging to the TNF superfamily, acts as a decoy receptor for RANKL, reducing its interaction with RANK and consequently suppressing osteoclastogenesis. Sr that binds to CaSR enhances the expression of OPG, which in turn suppresses osteoclasts. Sr also inhibits bone resorption by enhancing the apoptosis of mature osteoclasts [101].

Due to their common origin from MSCs, there is always a dilemma in determining the cell fate of endothelial cells and osteoblasts. In particular, aging disrupts the balance of differentiation by favoring the differentiation of MSCs into the adipogenic lineage rather than the osteogenic lineage. Increased levels of ROS accelerate the differentiation of MSCs into adipocytes. The signaling pathway tightly regulates this cascade, with CCAAT/enhancer-binding proteins (C/EBPs) and peroxisome proliferator-activated receptor γ (PPARγ) promoting adipogenesis, while TAZ and Runx2 pathways drive osteogenesis. Sr enhances the osteogenic differentiation of MSCs by exhibiting antioxidative properties, thereby increasing osteogenic differentiation and reducing adipogenic differentiation [94,95].

Considering the significant effect of Sr on bone regeneration, it has garnered increased attention in the field of dentistry. Periodontitis, a major cause of bone loss in the oral cavity, has led to increased interest in Sr-releasing biomaterials. These biomaterials have been proven to enhance the expression of ALP, bone matrix synthesis, and various osteogenic genes such as osteocalcin (OCN), Runx2, bone sialoprotein (BSP), and bone morphogenic protein (BMP) in pre-osteoblasts and osteoblasts [102]. Osteoblasts originate from bone marrow mesenchymal stem cells (BMSCs), and thus the commitment of BMSCs and MSCs located in the periodontal ligament to differentiate into osteoblasts plays a key role in bone regeneration. Novel tissue engineering scaffolds have incorporated Sr into mineralizing scaffolds to promote osteogenesis. This section discusses the Sr-incorporated biomaterials that have demonstrated enhanced bone regeneration in osteoblasts, BMSCs, and PDL stem cells.

The abundance of HA in the bone matrix has led to the widespread use of HA as the primary material in most bone tissue engineering scaffolds. This is because HA can mimic the extracellular matrix (ECM) of the bone. Derivatives of HA ceramics, including ɑ-tricalcium phosphate, biphasic calcium phosphate (BCP), and β-tricalcium phosphate cement, have been shown to possess excellent biocompatibility and bioactivity [103]. However, these materials alone are not sufficient to stimulate an osteogenic effect on osteoblasts or mesenchymal stem cells (MSCs). To overcome the limitations of HA-based synthetic bioceramics, such as bioactive glass, Sr is incorporated into them to enhance the osteogenic activity. Metal ions like magnesium (Mg), zinc (Zn), and Sr are naturally present as trace elements in the bone matrix. Sr can actively substitute Ca in the crystal lattice of HA, thereby enhancing the solubility and degradability of the bioceramics. Sr-substituted bioceramics have been developed in various forms, such as powders, granules, fibers, and three-dimensional (3D) scaffolds.

Tsai et al. [104] developed a nanofiber using Sr-substituted HA. It is widely recognized that cell interaction with nanofibers is more efficient compared to compact powder-based fillings. The mesoporous structure and high surface area of the electrospun scaffold facilitate better cell absorption and attachment. The Sr-substituted hydroxyapatite nanofibers (Sr-HNAFs) exhibited excellent drug loading efficiency and sustained release of the antimicrobial drug (tetracycline). Additionally, the Sr-HNAFs also enhanced the osteogenic differentiation of MG63 osteoblast-like cells by increasing ALP activity. The alignment of the nanofibers also influenced cellular behavior, with the aligned nanofibers of Sr-HA showing higher osteoblast inductive efficacy compared to randomly substituted Sr-HNAFs [105,106].

While there are several biomaterials that incorporate Sr, the field of dentistry primarily focuses on utilizing Sr-coated dental implant surfaces. Titanium (Ti) implants are considered the ideal scaffold materials for dental and orthopedic implants due to their excellent mechanical properties and biocompatibility. By coating or incorporating Sr ions onto the implant surface, the osseointegration of localized precursor cells can be enhanced.

## 8. Strontium and Implant Coatings

Titanium, its alloys, and zirconia are widely used biomaterials for orthopedic and dental implants due to their excellent biocompatibility, mechanical properties, and corrosion resistance [107]. However, these materials are biologically inert, which can lead to a prolonged healing period after surgery. Achieving early osseointegration and implant fixation is essential for ensuring long-term implant stability and reducing the duration of clinical treatment.

Surface modification techniques have been developed to improve osseointegration and accelerate the healing time for titanium and zirconia implants [108]. Various methods, such as the sandblasted and acid-etched (SLA) process, sol–gel process, plasma spraying, and chemical vapor deposition, have been employed for dental implant coating [109,110]. These coating methods can induce morphological changes, such as increased surface roughness, and modify the chemical composition by facilitating the formation of hydroxyapatite formed on the implant surface.

Bioactive materials like hydroxyapatite and bioactive glass are frequently used to modify the surfaces of titanium and zirconia implants. These materials exhibit remarkable biocompatibility and bioactivity. Hydroxyapatite possesses a chemical composition and structure similar to human bones and teeth, enabling it to form a strong chemical bond with bone tissue cells [111,112,113]. The application of these coating materials can greatly enhance the performance of titanium and zirconia implants.

The substitution of Ca with Sr in hydroxyapatite and bioactive glass implant coatings has been found to enhance their physical, mechanical, and biological properties [114,115]. This substitution has been shown to improve the osseointegration and bone healing of these coated implants [116,117]. In vitro studies have demonstrated that Sr-substituted hydroxyapatite and bioactive glass coatings promote the proliferation and differentiation of osteoblast cells, while also inhibiting the production and proliferation of osteoclasts [118,119]. In vivo studies have shown that Sr-substituted hydroxyapatite and bioactive glass coatings enhance new bone formation, implant fixation, and bone-to-implant contact during the healing period [120,121,122] (Table 3).

Recently, bioactive Sr-functionalized coatings on titanium and zirconia implants have been developed using two different techniques: micro-arc oxidation and magnetron sputtering [123,124]. One example of such a coating is the nanoporous micro-arc oxidized Sr–titanium (MAO-Sr-Ti) coating on titanium, which is fabricated through electrochemical surface treatment [123]. In this process, titanium undergoes micro-arc oxidation, and then Sr ions are incorporated into the surface through electrochemical treatment using a Sr dichloride solution. The resulting nanoporous structure increases stem cell adhesion, while the MAO-Sr-Ti coating promotes the proliferation and differentiation of bone marrow-derived mesenchymal stem cells. Furthermore, the coating facilitates early osseointegration and the formation of new bone.

Another example of a Sr-functionalized coating is the development of a Sr–titanate nanocoating on zirconia implants using magnetron sputtering [124]. In this process, the zirconia surface is first treated by sandblasting and acid-etched with hydrofluoric acid (SA-Zr). Then, Sr titanate is deposited onto the SA-Zr surface using magnetron sputtering. The resulting nanocoating enhances the spreading and adhesion of pre-osteoblast cells. It also promotes the proliferation and differentiation of osteoblasts, leading to improved osseointegration compared to the surface treated with sandblasting and acid-etching alone.

**Figure 5:**
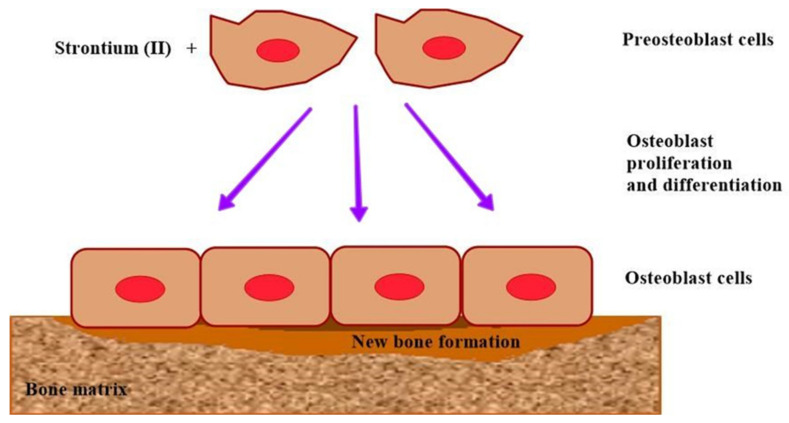
Schematic diagram of strontium’s effect on bone formation.

Indeed, Sr has been proven to enhance bone growth and regeneration by increasing the proliferation and differentiation of osteoblasts [125] (Figure 5). Additionally, Sr has the ability to inhibit apoptosis in osteoblast cells. Furthermore, Sr has been found to inhibit the formation and differentiation of osteoclast cells and promote their apoptosis, leading to a reduction in bone resorption [126]. Therefore, the incorporation of Sr in functionalized coating can significantly improve the biological properties of titanium and zirconia implants.

**Table 3:** Sr-doped biomaterials and implants with enhanced osteogenic differentiation of BMSCs, PDL stem cells, and osteoblasts.

Materials	Material Characteristics	Biological Characteristics	Reference
Sr-incorporated HA (SrHA)	- SrHA bone cement was developed in an injectable form - Compressive strength was 73.4 MPa - Favorable injectability (100%) - Setting time (initial setting time 240 s and final (420 s) - Excellent radiopacity	- Excellent biocompatibility - Promoted osteogenic differentiation of PDL stem cells and dental pulp stem cells (DPSCs)	Dai et al., 2021 [127]
	- Micro–nano hybrid surfaces doped with different concentrations of Sr (Sr–mnHAP)	- Promoted attachment of BMSCs to scaffold and enhanced osteogenic differentiation through upregulation of ALP and OCN - Promoted bone regeneration in rats with calvaria defects and enhanced vascularization	Jiang et al., 2022 [98]
SrHA with natural and synthetic polymers	- SrHA–SF biocomposite nanospheres - Mechanical strength was not calculated	- Enhanced osteogenic property of BMSCs	Wang et al., 2020 [97]
	- SrHA nanofibers synthesized with PVP, PCL, and PLLA	- Electrospun nanofibers showed extensive cellular homing for MG63 osteoblast-like cells - Enhanced osteogenic activity of MG63 cells	Tsai et al., 2018 [104]
Sr with bioactive glass	- Sr-incorporated mesoporous bioactive glass (Sr–BG)	- Periodontal defects were created through bilateral ovariectomy (OVX) - The filled periodontal defects with Sr–BG showed a 46.67% increase in bone formation when compared to BC scaffold alone - Increased expression of epigenetic regulator Setd2 in Sr–GB-induced bone formation	Jia et al., 2017 [94]; Zhang et al., 2014 [128]
Sr incorporation on dental implants	- Calcium–strontium–zinc phosphate coating on the Ti implant	- Enhanced osteogenic-related factors - Polarized the macrophages from M1 to M2 phase - Effects on PDL stem cells have not been explored	Zhao et al., 2021 [129]
	- Sr-doped titanium via sandblasted, large-grit, and acid-etching Sr-SLA	- Improved osseointegration and differentiation of BMSCs - Reduced intracellular ROS level, favorable for osteogenesis - Inhibited adipogenic differentiation - Enhanced anti-inflammatory properties	Zhou et al., 2019 [130]; Choi et al., 2018 [131]
	- Ti implant releasing both Sr and silver (Ag) ions	- Incorporation of Ag did not affect the effect of Sr on osteogenic property - Ag exhibited antibacterial property	Okuzu et al., 2021 [132]
	- Strontium-functionalized grade 4 Ti implants - Sustained release of strontium	- Assessed on rabbit femur model, showed increased bone-to-impact contact - No difference in % bone formation	Offermanns et al., 2018 [133]
	- Polyetheretherketone implant decorated with Sr and adiponectin	- Increased osteogenic activity of MC3T3-E1 cells - Adiponectin increased osteogenic properties - Sr and adiponectin had a synergistic effect on osteogenic property	Wang et al., 2019 [134]
	- Polybutylcyanoacrylate (PBCA) loaded with Sr, synthesized by emulsion polymerization methods.	- Higher expression of bone matrix deposition when treated on porcine mandibular bone block - Increased osteogenic differentiation of MSCs	Chang et al., 2021 [135]
Sr with hormone	- Parathyroid hormone (PTH) and Sr-containing poloxamer implant tablets	- PTH enhanced bone and antiresorptive molecule; Sr enhanced bone formation - PTH alone promoted bone formation in 4 weeks and declined after 3 weeks - Sr alone had no positive effects on bone formation - PTH + Sr showed significantly higher bone formation	Goker et al., 2018 [136]

## 9. The Influence of Strontium on Mechanical Properties

The mechanical properties of dental materials are crucial for ensuring durability, functionality, and biocompatibility in oral environments. They directly impact the long-term success of dental treatments and patient comfort. The incorporation of strontium into dental materials significantly enhances their mechanical properties through various mechanisms, including grain refinement and structural modification [137,138]. Research has shown that the inclusion of strontium in dental composites and adhesives can enhance their mechanical properties, such as flexural strength and wear resistance [139]. This improvement is attributed to the ability of strontium to reinforce the composite matrix and promote better bonding between the filler and the resin matrix. Arepalli et al. [137] investigated the effects of strontium substitution in bioactive glasses. Their study demonstrated that strontium substitution led to enhanced bioactivity, biocompatibility, and mechanical behavior of bioactive glasses. The incorporation of strontium improved the compressive strength and fracture toughness, making the material more suitable for dental applications. Another study investigated the mechanical properties of strontium ion-doped mesoporous bioactive glass. Their findings revealed that the doping of strontium ions significantly improved the compressive strength and elastic modulus of the mesoporous bioactive glass [140]. The Sr^2+^ in the glass phase serves as a network modifier, where it enhances the degree of cross-linking compared to unmodified cements. This suggests that the inclusion of the larger Sr^2+^ ion in the system amplifies the disturbance within the glass network. Consequently, a decrease in interatomic spacing causes the densification of Sr-1. This then leads to greater compactness within the glass structure, potentially contributing to an increase in elastic moduli [137]. On the other hand, the incorporation of Sr into calcium silicate-based materials did not favor the enhancement of the mechanical properties [141]. With the addition of Sr and gradual increase in concentration, the calcium silicate network might experience further loosening. This happens as the larger Sr^2+^ ions progressively replace the smaller calcium ions, causing a linear increase in lattice constants. This leads to considerable disorganization and a weakening of network connectivity [142]. The incorporation of strontium can have diverse effects on the mechanical properties of dental materials, depending on its interaction with the material structure. Hence, while strontium plays a crucial role in influencing the properties of dental materials, it is essential to carefully evaluate the mechanical properties and assess the potentially positive or negative impact of strontium on these properties.

## 10. Immunomodulatory Potential of Strontium

The host immune response plays a crucial role in the process of bone formation and homeostasis. This response involves a large number of cytokines, signaling molecules, and specific proteins that have a substantial role in immunomodulation and osteogenesis [143,144]. Many immune cells modulate and influence inflammatory responses. Macrophages are among the most effective cells in the immune system, as they play significant roles in the inflammatory response to external biomaterials [145]. There are two phenotypes of macrophages: M1 and M2. M1 macrophages have a pro-inflammatory effect, while M2 macrophages have anti-inflammatory potential and promote cellular proliferation and tissue repair [146]. Sr has been found to regulate the response of macrophages and suppress interleukin 6 (IL-6) [147].

Lee et al. [147] incorporated Ca and Sr in nanostructured titanium (Ti) surfaces and concluded that the chemically modified Ti disks with divalent cations regulated the cell shape of adherent macrophages and markedly upregulated the expression of the M2 macrophage phenotype when combined with the nanostructured Ti surface. The effect of Sr and Ca ions favored early wound healing by modulating the early M1 macrophage cells (after implantation of the nanostructured Ti implants). In another study, the immunoregulation potential of Sr-modified Ti surfaces using a novel phase transited lysozyme (PTL) was evaluated [148]. The Sr-modified group exhibited greater osteogenesis and controlled inflammation at the implant–tissue interface. Moreover, the expression levels of pro-inflammatory genes of macrophages on Sr-modified Ti implants were lower than on Ti implants without Sr [148]. When macrophages are activated into the M1 subtype, it increases the expression levels of pro-inflammatory factors, contributing to inflammation and foreign body reaction with subsequent fibrosis. On the other hand, when macrophages are activated towards the M2 subtype, they produce anti-inflammatory cytokines that promote healing and create favorable conditions for new tissue formation [149].

In a study conducted by Zhang et al. [150], the effect of Sr-substituted submicrometer bioactive glass (Sr–SBG) on macrophages was investigated. The interaction between Sr-SBG and macrophages both in vitro and in vivo was examined using histological assessment. Zhang et al. found that Sr–SBG enhanced osteogenesis and suppressed osteoclastogenesis. They attributed this effect to the stimulation of macrophage polarization from M1 to M2 by Sr–SBG, creating more suitable conditions for osteogenesis compared to SBG alone. Furthermore, the macrophages conditioned with Sr–SBG exhibited a significant decrease in IL-6 cytokine levels compared to the SBG group. This decrease may have contributed to the inhibition of osteoclastic activity. The inhibitory effect of Sr may be attributed to the downregulation of tumor necrosis factor α (TNF α) and the suppressed effect of NFkB, which hinders the differentiation of pre-osteoclasts.

Li et al. [151] developed a Sr–Cu borosilicate glass bone cement that could regulate bone healing by modulating inflammation, enhancing vascularization, and improving osteogenic differentiation of bone marrow-derived stem cells. They reported that the sustained controlled release of Sr and Cu ions upregulated anti-inflammatory genes (IL-1Ra and TGF-β1) while downregulating the expression of pro-inflammatory genes (IL-1β and IL-6) in macrophages. Several other studies incorporated Sr in different biomaterials, scaffolds, and coatings [129,131,152,153,154,155,156,157,158,159,160], and reported the same outcome, i.e., that Sr is capable of modulating macrophage polarization, which significantly promotes osteogenesis and suppresses osteoclastic activity. Interestingly, it can be theorized that the ability of Sr to induce bone formation and inhibit bone resorption may arise from its effect on modulating the macrophage inflammatory phenotypes.

## 11. Future Perspectives

Future research should focus on the regenerative potential of Sr and its role in alleviating reversible and irreversible pulp inflammation (pulpitis) by modulation of macrophages to improve the success of vital pulp therapies and dentin regeneration. Furthermore, more evidence is needed to validate the antimicrobial potential of strontium and its role in arresting dental caries.

The development of new bioactive strontium compounds such as strontium-based mixed-oxide ceramics can enhance biological performance, such as antimicrobial and bone regeneration properties [161,162].

## 12. Conclusions

The incorporation of strontium into dental biomaterials presents a significant advancement in the field of dentistry. Strontium enhances dental material visibility in radiographic imaging, supporting accurate diagnosis and treatment. It also exhibits antimicrobial properties that reduce secondary caries risk and improve restoration longevity. Its ability to promote enamel remineralization offers potential for managing dental caries and strengthening tooth structure. Though mixed results exist, strontium compounds have demonstrated effectiveness in addressing dentin hypersensitivity and potential for dentin regeneration, highlighting their potential in vital pulp therapies and regenerative dentistry. Strontium’s osteogenic effects make it promising for bone tissue engineering and dental implant biomaterials, with studies showing improved osseointegration and accelerated healing. Additionally, its immunomodulatory properties, particularly in macrophage polarization, contribute to favorable bone regeneration and healing environments. Strontium’s multifaceted benefits underscore its potential in advancing biomaterials that support tissue regeneration and modulate host immune responses for improved treatment outcomes.
